# Analysis of Chromosomal Aberrations and FLT3 gene Mutations in Childhood Acute Myelogenous Leukemia Patients

**DOI:** 10.5505/tjh.2012.24392

**Published:** 2012-10-05

**Authors:** Ender Coşkunpınar, Sema Anak, Leyla Ağaoğlu, Ayşegül Ünüvar, Ömer Devecioğlu, Gönül Aydoğan, Çetin Timur, Ahmet Faik Öner, Yıldız Yıldırmak, Tiraje Celkan, İnci Yıldız, Nazan Sarper, Uğur Özbek

**Affiliations:** 1 İstanbul University, Institute of Experimental Medical Research, Department of Genetics, İstanbul, Turkey; 2 İstanbul University, School of Medicine, Department of Pediatric Hematology-Oncology, İstanbul, Turkey; 3 Bakırköy Maternity and Children’s Hospital, İstanbul, Turkey; 4 Göztepe Education and Research Hospital, Department of Pediatric Hematology, İstanbul, Turkey; 5 Yüzüncü Yıl University, School of Medicine, Department Of Pediatrics, Van, Turkey; 6 Şişli Etfal Education and Research Hospital, Department of Pediatric Hematology, İstanbul, Turkey; 7 İstanbul University, Cerrahpaşa School of Medicine, Department of Pediatric Hematology-Oncology, İstanbul, Turkey; 8 Kocaeli University, School of Medicine, Department of Children’s Health and Diseases, Kocaeli, Turkey

**Keywords:** Childhood AML, FLT3 gene mutations, ITD, D835 mutations, Chromosomal translocations

## Abstract

**Objective:** To identify the well-known common translocations and FLT3 mutations in childhood acute myelogenousleukemia (AML) patients in Turkey.

**Material and Methods:** The study included 50 newly diagnosed patients in which t(15;17), t(8;21), and inv(16)chromosomal translocations were identified using real-time PCR and FLT3 gene mutations were identified via direct PCR amplification PCR-RE analysis.

**Results:** In all, t(15;17) chromosomal aberrations were observed in 4 patients (8.0%), t(8;21) chromosomal aberrationswere observed in 12 patients (24.0%), inv(16) chromosomal aberrations were observed in 3 patients (6.0%), and FLT3-ITD mutations were observed in 2 patients (4.0%); FLT3-D835 point mutation heterozygosity was observed in only 1patient (2.0%) patient.

**Conclusion:** Despite of the known literature, a patient with FLT3-ITD and FLT3-D835 double mutation shows a bettersurvival and this might be due to the complementation effect of the t(15;17) translocation. The reportedmutation ratein this article (4%) of FLT3 gene seems to be one of the first results for Turkish population.

## Abstract (tr)

**Amaç:** Bu çalışmada Türk çocukluk çağı AML hastalarında sık görülen kromozomal translokasyonların ve FLT3 mutasyonlarının belirlenmesi amaçlandı.

**Gereç ve Yöntemler: **Yeni tanı almış 50 hastada real time PCR yöntemi ile t(15;17), t(8;21), inv(16) kromozomal translokasyonlarının varlığı ve PCR yöntemi ile FLT3 geni ITD tipi mutasyonlar ve D835 nokta mutasyonları varlığı araştırıldı.

**Bulgular:** Olguların 4’ünde t(15;17) (8.0%), 12’sinde t(8;21) (24%), 3’ünde inv(16) (6.0%) kromozomal düzensizlikleri, 2 olguda FLT3-ITD mutasyonu (4.0%), 1 olguda FLT3-D835 nokta mutasyonu (2,0%) varlığı tespit edildi. t(15;17) pozitif AML M3’lü bir olguda hem FLT3-ITD hem de FLT3-D835 mutasyonları bakımından heterozigotluk tespit edildi.

**Sonuç:** Literatürden farklı olarak, bir hastamızın daha uzun sağkalımının t(15;17) translokasyonunun mutasyonlu bireylerde iyi yönde düzeltici etkisinin neden olabileceği düşünülmektedir. FLT3 geninde görülen bu mutasyon oranı (4%) Türk toplumu için ilk sonuçlardan biridir.

## CONFLICT OF INTEREST STATEMENT

The authors of this paper have no conflicts of interest, including specific financial interests, relationships, and/or affiliations relevant to the subject matter or materials included.

## INTRODUCTION

Acute myelogenous leukemia (AML) is a malignant disease of myeloid stem cells linked to oncogenic fusion proteins, which is due to chromosome translocations andinversions. Numerous translocations have been describedin AML, of which the most common are t(8;21), t(15;17),and inv(16). These recurring translocations are currentlyused as the basis for classification of AML [1]. As such,AML-associated fusion proteins function as aberrant transcriptionalregulators, with the potential to interfere withnormal myeloid cell differentiation [1,2,3]. FMS-like tyrosinekinase 3 (*FLT3*)—a new member of the receptor tyrosinekinase (RTK) III subfamily—was originally identifiedin hematopoietic stem/progenitor cells and is importantfor normal lymphohematopoietic stem cell function [4].*FLT3* is aberrantly expressed in the most of AML patients.The *FLT3* gene is located on chromosome 13 (13q12) [5].To date, 2 distinct types of *FLT3* gene mutations have beenidentified in AML cases: 1. Internal tandem duplication(ITD) mutations, which occur within the juxtamembraneregion of the gene; 2. Point mutations that occur at codon835 (D835) within the kinase domain. Both types of mutations constitutively activate *FLT3* tyrosine kinase activity[6]. *FLT3* gene mutations are strongly associated withleukocytosis and poor prognosis in AML patients [5,7,8].Patients with either of these mutations have a higher riskof recurrence and a lower survival rate [8]. It was recentlyreported that the *FLT3* gene mutant/normal ratio can beused as a marker for the selection of therapy [5,6,7,8]. The present study aimed to indentify the well-known common AML translocations and *FLT3* mutations in childhood AML patients in Turkey.

## MATERIALS AND METHODS

**Patients **

The study included 50 newly diagnosed childhood AML patients (28 male and 22 female) that presented for molecular diagnosis to Istanbul University, Institute of Experimental Medicine, Istanbul, Turkey, between October 2007 and July 2008. The Istanbul University, School of Medicine Ethics Committee approved the study protocol (project No. 1850/2007) and informed consent was provided by the patients’ parents. Diagnostic bone marrow samples were divided into 2 parts; 2 x 107 cells were preserved in RTL buffer (cat. No. 79216, Qiagen, Germany) at –80 °C until RNA isolation and the remainder of the samples were used for DNA isolation, according to the manufacturer’s instructions (cat. No. 11796828001 Roche Applied Sciences, Germany). 

**Determination of t(15:17), t(8:21) and inv(16) chromosome abnormalities **

Total RNA was extracted from bone marrow specimens using a QIAamp RNA Blood Mini Kit (cat. No. 52304, Qiagen, GmbH, D-40724 Hilden, Germany), according to the manufacturer’s instructions. cDNA was synthesized from 1 μg of total RNA, as previously described [7]. The quality of the obtained cDNA was evaluated via ß-globin PCR performed using the following primers: forward: 5’ GAA GAG CCA AGG ACA GGT AC 3’; reverse: 5’ CAA CTT CAT CCA CGT TCA CC 3’. Chromosomal abnormalities [t(15; 17), t(8; 21), and inv(16)] were identified via real-time PCR, using the LightMix primer/probe set (cat. No. 40-0135- 16 cat. No. 40-0196-16 cat. No. 40-0229-16 TIB Molbiol GmbH, Berlin, Germany), and the Light Cycler FastStart DNA Master Hyprobe Kit (cat. No. 03515575001, Roche Diagnostics, GmbH, Mannheim, Germany).

**Detection of *FLT3*-ITD mutations **

*FLT3*-ITD mutations were indentified via PCR. The forward primer was in exon 14 (14F 5’-GCAATTTAGGTATGAAAGCCAGC- 3’) and the reverse primer was in exon 15 (15R 5’-CTTTCAGCATTTTGACGGCAACC-‘3), as described by Wang et al. [4]. Amplification was performed in a reaction volume of 50 μL with 100 ng μL–1 of DNA, 10 pmol of each primer, 10 mmol dNTP, and 2.5 U of Ex-Taq DNA polymerase (cat. No. RR001A Takara, Japan) in the buffer (10 mmol L–1 of TrispHCl [pH 8.3], 50 mmol L–1 of KCl, and 1.5 mmol L–1 of MgCl2). The PCR conditions were as follows: initial denaturation at 95 °C for 5 min, 95 °C for 30 s, 60 °C for 30 s, and 72 °C for 30 s for 30 cycles, and elongation for 10 min at 72 °C. Amplification products were analyzed on 3% agarose gel stained with ethidium bromide and samples with the specific PCR products (329bp) were considered as positive for *FLT3*-ITD mutations. The specific amplicons were purified using the QIAEX II Gel Extraction Kit (cat. No. 20021, Qiagen, Hilden, Germany), according to the manufacturer’s instructions, and directly sequenced for confirmation of PCR. 

**Detection of *FLT3*-D835 mutations **

FLY3-D835 mutations were identified using the PCRRFLP method. The primers employed were 20F 5’-CGC Figure CAGGAACGTGCTTG-3’ and 20R 5’-GCAGCCTCACATTGCCCC- 3’, as described by Wang et al. [4]. At codon 835 an aspartate amino acid is encoded, providing a recognition site for restriction enzyme EcoRV; as such, mutants can be detected via the loss of this enzyme restriction site. The PCR setup was as described above. The specific products were detected on agarose gel, followed by EcoRV (cat. No. 50-720-3590 Takara, Japan) digestion at 37 °C for 4 h. Restriction products were detected on a 3.5% agarose gels and undigested PCR product indicated the presence of the mutation (Figure). The results were confirmed via direct sequencing. 

**Statistical analysis **

Clinical and laboratory characteristics at diagnosis were statistically correlated (age, sex, WBC count, hemoglobin level, PLT count, blast cells rate) with t(15;17), t(8;21), inv(16) chromosomal aberrations, and *FLT3* mutations (Table 1). Fisher’s exact test and Pearson’s chi-square test were performed using SPSS v.12.0. P values less than 0.05 were considered statistically significant.

## RESULTS

Diagnoses—based on French-American-British (FAB)classification—were as follows: M0 (n=3); M1 (n=9);M2 (n = Diagnoses—based on French-American-British (FAB) classification—were as follows: M0 (n = 3); M1 (n = 9); M2 (n = 15); M3 (n = 12); M4 (n = 3); M5 (n = 5); M7 (n = 1). Additionally, 1 patient was lacking clinical data and could not be classified, and another patient that died following BMT and couldn’t be classified was thought to15); M3 (n = 12); M4 (n = 3); M5 (n = 5); M7(n = 1). Additionally, 1 patient was lacking clinical dataand could not be classified, and another patient that diedfollowing BMT and couldn’t be classified was thought to be M0 or M7. Median age of the patients was 8.42 ± 5.24 years (range: 0-18 years). The median white blood cell (WBC) count was 30,394.17 ± 57,255.86 mL–1 (range: 890-260,000 mL–1), the median platelet (PLT) count was 83,851.06 ± 76,349.87 L–1 (range: 2 x 109-291 x 109 L–1), the median hemoglobin level was 3.4 ± 2.67 g dL–1 (range: 3.4-14.3 g dL–1), and the median bone marrow blast rate was 60.86% ± 22.9% (range: 12%-100%). 

FAB classification and clinical features of the 50 childhood AML patients are summarized in Table 2. In all, 4 patients were positive for t(15;17), 12 were positive for t(8; 21), and 3 were positive for inv(16) (Table 3). The 4 t(15;17)-positive patients were classified as M3, and 9 of the 12 t(8;21)-positive patients were M2, 2 were M1, and 1 was M4. Among the inv(16)-positive patients, 1 was M0, 1 was M1, and 1 was M5. None of the patients were classified as M6; therefore, statistical evaluation of the FAB M6 patients was excluded. In total, 2 patients had *FLT3* gene mutations, 1 of which was classified as AML-M3 and interestingly the *FLT3*-D835 mutation was not a deletion, but a point mutation (g.IVS20 +49 A>G) that also changed the EcoRV restriction site. Both of these changes were previously described and are known to increase expression of *FLT3* [1,5,6]. 

The hemoglobin level in the patients with *FLT3*-ITD mutations was significantly lower than in the patients without the mutation. In the present study there was a correlation between FAB M2 classification and t(8;21) positivity (P = 0.005), and between FAB M3 classification and t(15;17) positivity (P = 0.009), which is in agreement with previous reports. In addition inv(16) was positive in the FAB M0, M1, and M5 patients with P values of P = 0.001, P = 0.003, P = 0.002 respectively.None of the FAB M4 patients were positive. Bone marrow blast rates below and higher than 80% were compared with t(8; 21) positivity and found that patients with t(8;21) had higher blast rates than non translocated ones (P = 0.049).

## DISCUSSION

In addition to observation of the standard clinical features and laboratory analysis, the diagnosis of AML requires additional procedures, including pathological examination, immunophenotyping, cytogenetics examination, and molecular diagnostics. Identification of the specific cytogenetic abnormality is important for selection of appropriate therapy and prognostic analysis [1,9]. Numerous translocations have been described in AML, of which the most frequent are t(15;17), t(8;21), and inv(16), accounting for 20%-30% of all chromosomal aberrations [1,10,11,12].These aberrations, depending on their structure, lead to expression of a chimeric protein with new functions [8,13].Prognosis is considered goodin cases oft(15;17) proliferaassociated with AML-M3, t(8;21), inv(16) associated with AML-M2, and inv(16) associated with AML-M4 [3,14,15]. It is known that the blast level in AML-M2 patients is 30%- 90% [16,17,18].

In the present study the bone marrow blast rate in 11 of the 12 FAB M2 patients with t(8;21) transloc],[18]. In the present study the bone marrow blast rate in 11 of the 12 FAB M2 patients with t(8;21) translocation was over 80%, which shows that the bone marrow blast level in childhood AML patients classified as FAB M2 is high. Additionally, inv(16) was strongly correlated with FAB classification (P = 0.043). The 3 inv(16)-positive AML patients were classified as follows: M0 (n = 1); M1 (n = 1); M5 (n = 1). According to the literature, inv(16) occurs more frequently in patients classified as FAB M4 [19] and AML-M4 occurs more frequently in patients classified as FAB M4, primarily in patients aged ≥50 years [17,18]. The presence of inv(16) is indicative of a good prognosis in FAB M4 patients, but data concerning the relationship between inv(16) and other AML FAB classifications are lacking. In the present study inv(16) was not observed in any of the FAB M4 patients. The present findings are in agreement with those reported by Dash et al. and the American National Cancer Institute AML guideline [14,20].The *FLT3* gene is expressed primarily in hematopoietic stem cells [5,21,22]. Moreover, human leukemia and lymphoma cell lines also express *FLT3* protein [22]. The 2 most common mutations of the *FLT3* gene are *FLT3*-ITD and *FLT3*-D835. Among the 50 childhood AML patents in the present study, only 2 had *FLT3*-ITD mutations, of which 1 also had *FLT3*- D835 point mutation. The incidence of FTL3-ITD mutation in childhood AML patients (5-16%) is lower than that in adult AML patients (20-25%) [22,25]. Meshinchi et al. reported that the incidence of *FLT3*-TKD (tyrosine kinase domain) mutations in childhood AML patients was 6.7% [27], versus 7% reported by Karabacak et al. [23]. The known *FLT3* gene mutation rate in adult AML patients is 20%-25% [22,25]; however, Liang et al. [24] reported a mutation rate in childhood AML patients of 11.3%, and Kondo et al. [22], Iwai et al. [25], and Krstovski et al. (26) reported rates of 5.3%-16.5%.

Our data is one of the first results *FLT3*-ITD mutation evaluation in Turkish pediatric AML patients and the mutation rate is 4.0%, which is much lower than the other study groups [23,27]. In the present study there wasn’t a correlation between *FLT3* gene mutations, and age, gender, the WBCcount, blast cell rate, or FAB classification. Although it was reported that the *FLT3*-ITD mutation rate increases with age Meshinchi et al. [27], Kondo et al. [22] and Wang et al. [4] and was not observed a significant difference between the mutation rate and the age in our results.

In the present study there wasn’t a significant difference between *FLT3*-ITD mutations and the WBC count. The WBC count in 95% of the present study’s patients was less than 50 x 109 L–1, which is in agreement with the findings reported by Moreno et al. [28]. The most common feature of AML is chronic, severe anemia, which damages bone marrow. The present results show that there was a relationship between a low hemoglobin level and *FLT3*-ITD mutations, but not between a low hemoglobin level and *FLT3*-D835 mutation, which might have been due to the small number of patients with *FLT3*-D835 point mutation as well as the small study population. Pre-clinical studies highlighted the potential use of inhibitors against *FLT3* kinase activity. Most of patients carrying *FLT3* gene mutation have a 50% decrease in the peripheral blast count, along with inhibition of receptor autophosphorylation.

Herein we described the development of anemia in patients carrying *FLT3* gene mutation. It is also known that *FLT3* mutations are poor prognostic markers of AML. A 7-year-old male patient in the present study classified as AML-M3 had double *FLT3* mutations and t(15;17) translocation. He was in remission for 1 year, and died after 19 months of diagnosis. While *FLT3* mutation causes continuous tyrosine kinase activity, t(15;17) translocation deregulates RARα transcription factor and cell differentiation stops. Thus, AML occurs due to the combined effects of *FLT3* gene mutations and t(15;17) translocation. To the best of our knowledge our 7-year-old male patient is the first reported case to have been in remission for approximately 1 year with double *FLT3* mutation and translocation. This is also the first case with double *FLT3* mutation and t(15;17) translocation that is reported inTurkish population. Most *FLT3* gene mutations are reported as individual mutations and only a few cases of double mutation have been reported [5,22,29,30]; Moreno et al. reported 4 cases in 2003 [28] and Wang et al. reported 2 cases in 2005 [4]. All the reported double mutation patients died after induction therapy or relapsed in first months after diagnosis [28]. These results indicate that *FLT3*-ITD and *FLT3*-D835 mutations are markers of poor prognosis. In contrast to previous reports our 7-year-old patient survived longer, which might have been due to the combined effect of t(15;17) translocation (31,32); however, the quality of clinical response to *FLT3* inhibitors has been minor, with many patients transiently responding with a decreased blast count(31,32). This finding needs to be confirmed by in vitro studies in which patients are followed-up for longer periods of time. It is not clear if *FLT3* double mutation causes upregulation in tyrosine kinase activity or increases cell survival. AML occurs as a result of excessive prolifera tion and differentiation of myelogenous blasts. The present study is the first to perform detailed molecular analysis of Turkish childhood AML patients. The low *FLT3* gene mutation rate (4%) seems to be unique to this study’s population. This result and the effects of double mutations need to be evaluated in larger patient groups.

We wish to thank the Research Council of Istanbul University for supporting this study.

## Figures and Tables

**Table 1:**
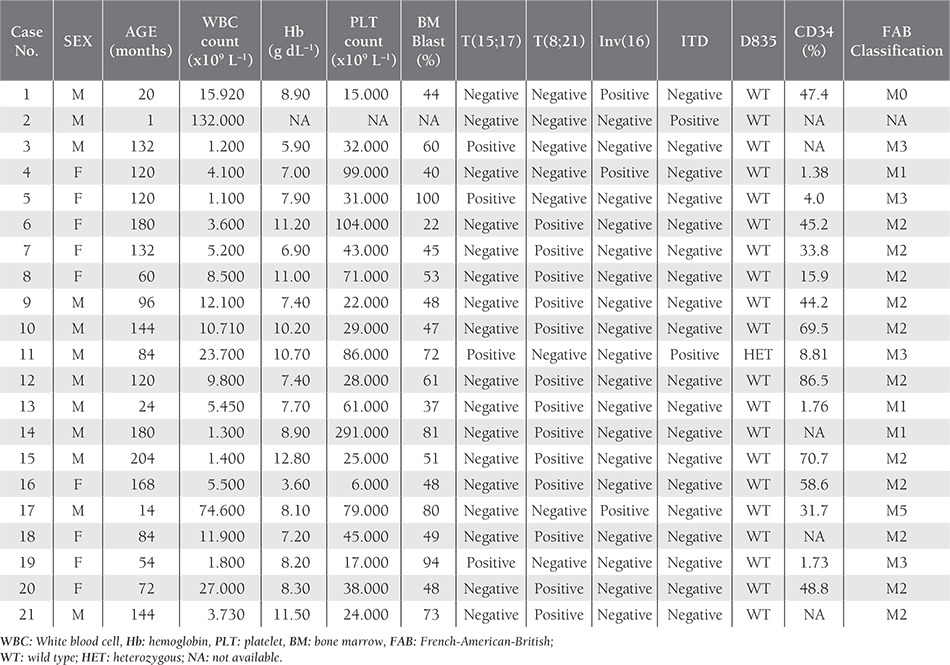
Characteristics of childhood AML patients carrying *FLT3* gene mutations and/or chromosomal aberrations.

**Table 2:**
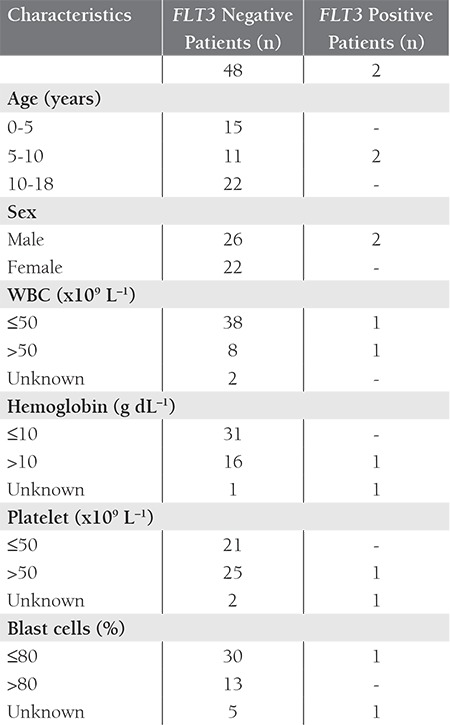
Patient mutation status and clinical features.

**Table 3:**
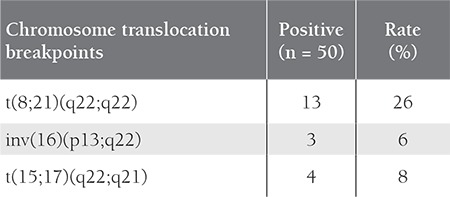
The frequency of translocations in the childhood AML patients.

**Figure 1:**
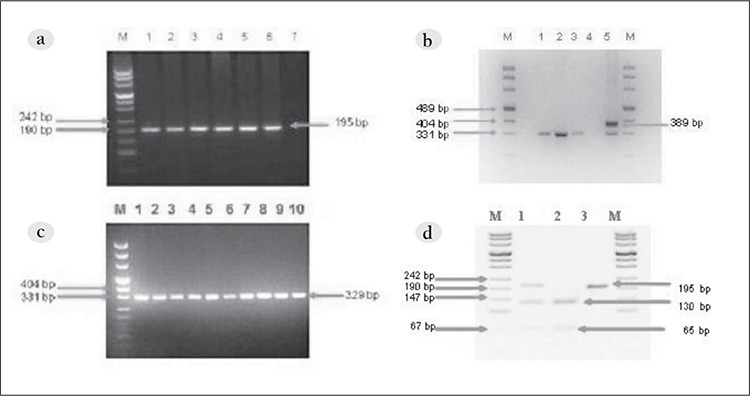
a. PCR amplification of the *FLT3* -ITD region (lane M: size marker; lanes 1-10: normal samples). b. PCR amplification of the *FLT3* -ITD region (lane M: size marker; lanes 1-3: normal samples; lane 4: negative control; lane 5: *FLT3* /ITD-positive case).c. PCR amplification of *FLT3* -D835 (lane M: size marker; lanes 1-6: normal samples; lane 7: negative control). d. D835 mutation detection (lane M: size marker; lane 1; *FLT3* -D835-positive case; lane 2: wild type; lane 3: EcoRV undigested sample).
